# Comparative evaluation of MTA and PRF for direct pulp capping in permanent mandibular molars - A randomized control trial

**DOI:** 10.6026/973206300221802

**Published:** 2026-03-31

**Authors:** Niharika Soni, Poonam Bogra, Swati Chhabra, Nikita Goyal, Himani Arora, Pooja Bala

**Affiliations:** 1 Department of Conservative Dentistry and Endodontics, J.N. Kapoor DAV Centenary Dental College and Hospital, Yamunanagar, Haryana, India

**Keywords:** Direct Pulp Capping (DPC), Electric Pulp Testing (EPT), Mineral Trioxide Aggregate (MTA), Platelet Rich Fibrin (PRF), Sodium Hypochlorite (NaOCl)

## Abstract

Direct pulp capping (DPC) aims to encourage pulp healing and the development of a mineralized tissue barrier and its successful implementation prevents the need for extensive treatment by protecting the exposed pulp with a dental biomaterial. Therefore, it is of interest to evaluate and compare the clinical outcome, radiographic outcome, pain score and analgesic intake after Direct Pulp Capping (DPC) using Mineral Trioxide Aggregate (MTA) and Platelet Rich Fibrin (PRF) as pulp capping agents in cariously exposed permanent mandibular molars with reversible pulpitis. Hence, a total of 154 permanent mandibular molars following the inclusion exclusion criteria were selected for the study. Teeth were randomly divided into two groups (Group I and II) of 77 teeth each. DPC was done with MTA and PRF in groups I and II respectively. All the samples were then evaluated at 1, 3, 6 and 12 months postoperatively. On statistical evaluation a significant difference was found between Group I (MTA) and Group II (PRF) at 6 and 12months follow-up period. PRF showed a higher success rate and less postoperative pain than MTA at all intervals, indicating its strong potential as a superior direct pulp capping material.

## Background:

Root canal treatment (RCT) is a reliable option for managing pulpal pathologies; however, recent evidence suggests that DPC is effective in managing deep carious lesions with reversible pulpitis [1]. DPC involves the placement of a biocompatible material directly over an exposed vital pulp due to caries, trauma, or operative procedures to promote healing and dentin bridge formation [2]. Historically, calcium hydroxide has been the material of choice due to its ability to stimulate reparative dentin. However, the resulting dentin bridge is often porous and insufficiently seals the pulp, leading to a high risk of failure [3, 4]. To address this, Mineral Trioxide Aggregate (MTA), a bioceramic material has been widely adopted [5]. MTA demonstrates superior biocompatibility, bioactivity and sealing ability and stimulates dentinogenesis via progenitor cell differentiation [6]. However, drawbacks such as discoloration, toxicity due to presence of arsenic, long setting time, difficult handling and higher cost limit its use [5]. Recently, biological alternatives like Platelet-Rich Fibrin (PRF) have been explored. PRF, a second-generation autologous platelet concentrate, promotes stem cell recruitment, angiogenesis and mineralization through the sustained release of growth factors [7]. Therefore, it is of interest to evaluate the clinical and radiographic outcomes of MTA and PRF as direct pulp capping agents in cariously exposed permanent mandibular molars with reversible pulptitis.

## Materials and Methodology:

This randomized controlled trial was conducted to evaluate and compare the effectiveness of MTA and PRF as Direct Pulp capping agents. The procedures followed were in accordance with the ethical standards of the responsible committee on human experimentation (institutional or regional) and with the Helsinki Declaration of 1975, as revised in 2000 Ethical approval was obtained from the Institutional Ethical Committee (Reference no. F/IEC/23/0298). Written informed consent was obtained from the participants prior to investigation. Patients aged 18 to 50 years with permanent mandibular first or second molars exhibiting deep occlusal caries and diagnosed with reversible pulpitis were included in the study. Only systemically healthy individuals without any radiographic signs of periapical pathology were selected. Teeth had to be restorable with composite resin and amenable to rubber dam isolation. Exclusion criteria included teeth diagnosed with irreversible pulpitis or necrosis, those with clinical signs like tenderness on percussion, sinus tract, mobility, or periapical radiolucency, pregnant or lactating women, patients with NSAID allergies and teeth where pulp was not exposed on caries excavation or bleeding could not be controlled within 10 minutes using 3% sodium hypochlorite. Based on a reported 85% success rate for MTA¹ and an expected 15% relative difference for PRF (≈13% absolute difference), the sample size was calculated with a 5% significance level (two-tailed) and 80% power. After adjusting for a 10% loss to follow-up, the final sample size was 77 participants per group. A total of 154 teeth diagnosed with reversible pulpitis were randomly allocated into two groups (Group I and Group II) of 77 teeth each using a computer-generated randomization sequence. After administering local anaesthesia, isolation was achieved using rubber dam and the tooth was disinfected with 2% chlorhexidine. Caries removal was done under 3x magnification using carbide burs and spoon excavators with the help of caries detector dye. Haemostasis was achieved using 3% sodium hypochlorite soaked cotton pellet. If haemostasis was not achieved within 10 minutes, the tooth was excluded from the study. In Group I, MTA Angelus was mixed with distilled water and placed over the exposure site, followed by temporary sealing. After setting of the cement, the cavity was lined with GIC and restored using nano-ceramic composite resin. In Group II, A-PRF Plus was prepared from 10 ml of venous blood via centrifugation (1300 rpm, 8 min) and placed over the exposure. The cavity was then restored similarly with RMGIC and composite. Patients were advised to take Piroxicam 20 mg for pain if needed and patients were asked to record pain scores on VAS scale. RCT was performed in cases of unresolved symptoms. Clinical and radiographic follow up examination was done at 4 days, 1, 3, 6 and 12 months. On clinical evaluation spontaneous pain/ referred pain, post-operative sensitivity greater than 15 seconds, tenderness on percussion and loss of pulp vitality were considered as failure. On radiographic evaluation periapical radiolucency or PDL widening were considered as failure. Post-operative pain evaluation was done after 6, 12, 24 hours, 2 and 3 days. Analgesics consumed during 3 days postoperatively were recorded on the 4th day.

## Statistical evaluation:

Descriptive statistics was performed by calculating mean and standard deviation for the continuous variables. Categorical variables were presented as absolute numbers and percentage. The success rates were compared using chi-square test. The pain scores and analgesic intake were compared using Mann-Whitney U test.

## Results:

The clinical success rates were found to decrease from 90.1% in Group I (MTA) and 97.4% in Group II (PRF) at 4 days to 67.5% in Group I and 85.7% in Group II at 12 months. A significant difference was found between Group I and Group II at 6 and 12 months (p<0.05) (Table 1 - see PDF). On radiographic evaluation success rates were found to decrease from 98.7% in Group I and 100% in Group II at 4 days to 93.5% and 97.4% in Group I and II respectively at 12 months. There was no significant difference found between the two groups (p>0.05) (Table 2 - see PDF).

## Discussion:

Direct Pulp Capping (DPC) offers a conservative, minimally invasive alternative aimed at preserving pulp vitality [8]. Permanent mandibular molars were chosen due to their larger pulp volume and better collateral circulation, which enhance healing potential and contribute to higher success rates [9]. When compared to Group I, Group II consistently showed higher success rates, with statistically significant differences at 6 and 12 months (p = 0.04). The observed differences in outcomes between MTA and PRF can be attributed to their distinct mechanisms of action as MTA promotes dentin bridge formation through reparative biochemical pathways, while PRF, as a biologically active scaffold, supports true pulp tissue regeneration via the sustained release of autologous growth factors [7]. MTA, despite being effective, has limitations such as initial cytotoxicity due to its high pH, handling difficulties and compromised sealing ability due to calcium hydroxide release during setting [9]. In contrast, PRF is fully biocompatible and autologous, with no cytotoxicity [10, 11]. PRF contains platelets and leukocytes within a fibrin matrix, releasing key growth factors (e.g., TGF-β1, VEGF, PDGF, EGF, IGF-1) and cytokines (IL-1β, IL-6, IL-4, TNF-α), which play vital role in pulp healing, angiogenesis and tissue regeneration [12, 13]. PRF's anti- inflammatory effects, facilitated by leukocytes, cytokines and fibrin, contributed to faster pain reduction. MTA has been shown to have limited antimicrobial activity, potentially impacting its healing performance [14, 15]. According to Tiwari *et al.* PRF when compared with MTA, is a suitable substitute material for DPC of primary teeth and can be classified as a promising therapeutic biomaterial [16]. However, limitations of the study include the inability to blind the operator, a relatively short 12-month follow-up, lack of CBCT or histologic analysis and reliance on subjective bleeding time for pulp assessment. Future studies with larger sample sizes, longer follow-up and advanced diagnostics are needed to validate these findings.

## Conclusion:

The study comparing PRF and MTA for direct pulp capping revealed that PRF achieved significantly higher success rates at both 6 and 12 months. Patients treated with PRF also reported significantly less postoperative pain and less analgesic intake at all evaluated time intervals compared to those treated with MTA. These findings indicate that PRF not only provides superior clinical and patient-centered outcomes but also shows a strong potential to serve as a promising alternative to MTA, the current gold standard for direct pulp capping. Thus, advancement in knowledge regarding pulpotomy is the shift towards regenerative biomaterials to promote the healing of remaining radicular pulp.
